# Efficacy of exercise-based prehabilitation for patients undergoing elective spinal surgery: a systematic review and meta-analysis

**DOI:** 10.3389/fmed.2025.1707138

**Published:** 2025-11-19

**Authors:** Lian-song Lu, Shao-hua Sun, Hao-jie Li, Zhen-shan Yuan

**Affiliations:** 1 Department of Spinal Surgery, Ningbo No.6 Hospital, Ningbo, Zhejiang, China; 2 Ningbo Clinical Research Center for Orthopedics, Sports Medicine & Rehabilitation, Ningbo, Zhejiang, China

**Keywords:** exercise-based prehabilitation, elective spinal surgery, preoperative outcomes, postoperative recovery, meta-analysis

## Abstract

**Background:**

The study aimed to investigate the efficacy of exercise-based prehabilitation for preoperative and postoperative outcomes in patients undergoing elective spinal surgery.

**Methods:**

A total of five databases were searched from their inception to March 2025 with no date restrictions. Standardized mean differences (SMDs) and mean differences (MDs) with 95% confidence intervals (CIs) were pooled using random effects models. The certainty of the evidence was assessed using the Grading of Recommendations, Assessment, Development, and Evaluation (GRADE) approach.

**Results:**

In total, six studies with 365 participants were included in this study. Preoperative (after prehabilitation): Exercise-based prehabilitation produced modest improvements compared to controls for back pain (SMD −0.32, 95% CI −0.54 to −0.11; *I*^2^ = 0%; GRADE: moderate), leg pain (SMD −0.43, −0.79 to −0.08; *I*^2^ = 53%; GRADE: moderate), knee extensor strength (SMD 0.33, 0.07 to 0.58; *I*^2^ = 0%; GRADE: moderate), disability (SMD −0.44, −0.65 to −0.23; *I*^2^ = 0%; GRADE: moderate), kinesiophobia (SMD −0.30, −0.53 to −0.07; *I*^2^ = 0%; GRADE: moderate), and depressive symptoms (SMD −0.24, −0.47 to −0.01; *I*^2^ = 0%; GRADE: moderate). Health-related quality of life (HRQoL) favored prehabilitation, but the CI included no effect (SMD 0.51, −0.04 to 1.07; *I*^2^ = 71%; GRADE: moderate). Postoperative short-term results (≤1 month): Early back pain improved (SMD −0.51, −0.93 to −0.08; *I*^2^ = 36%; GRADE: moderate). One trial reported improved short-term HRQoL. Moreover, length of hospital stay (days) was shorter but not statistically significant (MD −1.30 days, −2.89 to 0.29; *I*^2^ = 77%; GRADE: low). Other short-term, intermediate-term (1–6 months), and long-term (≥6 months) results: Pooled estimates for back pain, leg pain, disability, kinesiophobia, depression, and HRQoL clustered near the null, with moderate-certainty evidence for most outcomes and no consistent durable benefit.

**Conclusion:**

Exercise-based prehabilitation provides consistent small-to-moderate standardized benefits for most preoperative outcomes and shows a favorable signal for early postoperative back pain in adults undergoing elective spinal surgery. However, evidence for sustained intermediate- and long-term postoperative improvements is not established with current data.

**Systematic Review Registration:**

CRD420251120535, https://www.crd.york.ac.uk/PROSPERO/view/CRD420251120535.

## Introduction

Degenerative spinal disorders, including low back conditions, represent a significant global cause of disability, placing an increasing burden on patients and healthcare systems worldwide (1). Elective spinal surgeries, such as decompression, discectomy, and instrumented fusion, are frequently performed, contributing substantially to surgical workload and healthcare expenditures in both high- and middle-income countries (2). Despite advancements in surgical techniques and perioperative care, a clinically significant subset of patients continues to experience persistent postoperative pain, disability, or “failed back surgery syndrome” (3), resulting in long-term morbidity and resource utilization (4). Several modifiable preoperative factors, including reduced cardiorespiratory fitness, weakness in limb and paraspinal muscles, nutritional deficiencies, tobacco use, and untreated psychological distress (such as depression, catastrophizing, and kinesiophobia), are associated with poorer postoperative outcomes across surgical populations and represent plausible targets for preoperative optimization in spine patients (5). Psychological constructs, particularly fear-avoidance and kinesiophobia, are linked to higher baseline pain and disability and may limit engagement with rehabilitation, thereby increasing the risk of poor postoperative recovery (6).

Prehabilitation, defined as structured, time-limited programs implemented before elective surgery to enhance physical capacity, nutritional status, and psychological readiness, possesses a strong theoretical foundation for elective spinal procedures and has demonstrated improvements in preoperative function and physiological reserve in other surgical domains (7, 8). Exercise-based prehabilitation specifically targets the enhancement of muscle strength and aerobic capacity while aiming to reduce fear-avoidant behaviors, changes that are theoretically expected to facilitate earlier mobilization, enhance participation in postoperative rehabilitation, and decrease short-term complications and length of hospital stay (9–11). Nevertheless, the evidence supporting exercise-based prehabilitation in the context of elective spine surgery remains limited and heterogeneous. Randomized controlled trials (RCTs) conducted in this area have been small in scale, exhibit variability in the content and intensity of interventions, and employ inconsistent timing and outcome measures (12). Given the high population burden of spinal disease, the non-trivial incidence of persistent postoperative pain and disability, and the biological plausibility that improving preoperative physical and psychological readiness could alter postoperative trajectories, a focused, outcome-specific synthesis of randomized evidence on exercise-based prehabilitation in elective spine surgery is timely and necessary (13, 14).

To the best of our knowledge, no comprehensive systematic review with meta-analysis has concurrently evaluated the efficacy of exercise-based prehabilitation on preoperative and postoperative outcomes in patients undergoing elective spinal surgery. Accordingly, we performed a systematic review and meta-analysis of RCTs comparing exercise-based prehabilitation with non-exercise controls in adults scheduled to undergo elective spinal surgery to address this evidence gap and provide evidence-based recommendations for clinical practice and future research.

## Methods

**Figure 1:**
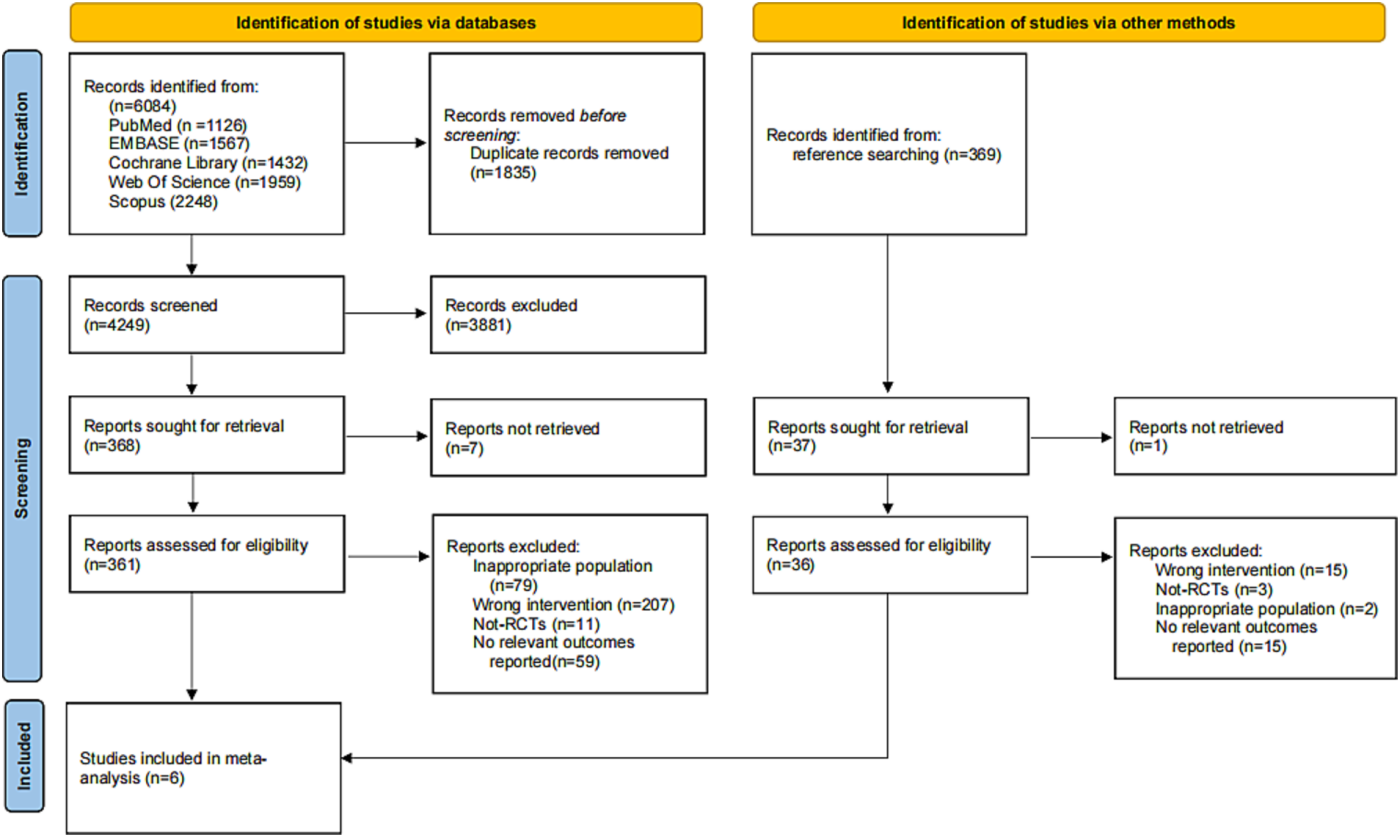
PRISMA flowchart depicting the study identification and selection process.

This systematic review was carried out following the methods of the Cochrane Handbook (15), according to the guidelines set by the Preferred Reporting Items for Systematic Reviews and Meta-Analyses protocols (PRISMA-P) (16). The protocol was registered on the International Prospective Register of Systematic Reviews (PROSPERO): CRD420251120535. The review process is illustrated in a flow diagram (Figure 1).

### Electronic searches and study selection

We conducted a comprehensive literature search in March 2025 to capture all potentially eligible trials, without restrictions on language or publication date. The following five online databases/sources were searched from their inception: PubMed, Embase, the Cochrane Central Register of Controlled Trials (CENTRAL), Scopus, and the Web of Science. The search strategy was developed and implemented by an independent medical librarian, with complete algorithms provided in Supplementary material S1. To ensure comprehensive coverage, forward and backward citation searching was conducted for all included studies. For full texts that were not accessible through institutional subscriptions or interlibrary loans, we contacted corresponding authors via email to request manuscripts or clarify unpublished data. All citations were imported into EndNote for de-duplication, after which two independent reviewers screened titles, abstracts, and full texts against predefined eligibility criteria. Discrepancies between the reviewers were resolved through discussions and consensus.

### Data extraction

A total of two reviewers independently performed data extraction following the Cochrane Handbook for Systematic Reviews of Interventions (version 6.3) guidelines. For each eligible trial, we recorded study characteristics (author, year, and country), participant demographics (age, gender, sample size), intervention and control groups details, and outcome measures. Eligible studies comprised randomized and quasi-randomized controlled trials evaluating the effectiveness of exercise-based prehabilitation versus non-exercise controls in patients undergoing elective spinal surgery. For each outcome, mean and SD (standardized difference) data were extracted for intervention and control groups at preoperative and postoperative time points, as well as for all other follow-up assessments. We divided outcomes into preoperative (after prehabilitation), postoperative short-term (≤1 month), postoperative intermediate-term (>1 to <6 months), and postoperative long-term (≥6 months) phases. If sufficient studies are available, data from different time points will be extracted for meta-analyses. Animal trials and non-English studies were excluded. Any disagreements were resolved by discussion or, if necessary, by consultation with a third reviewer.

### Data analysis

Pooled estimates of treatment effects for continuous outcomes were combined using either mean differences (MD) or standardized mean differences (SMD) with 95% confidence intervals (CIs). We preferentially applied a random effects model to account for between-study variability among trials judged to be clinically and methodologically comparable. Following the Cochrane recommendations, if means or standard deviations were not directly reported, we estimated them from available *p*-values, CIs, or standard errors. Following Cohen (17), effect sizes were interpreted as follows: large (≥0.8), moderate (0.5–0.8), small (0.2–0.5), and trivial (<0.2). Statistical heterogeneity was evaluated for each pooled analysis using the *I*^2^ statistic and categorized as follows: low (<25%), moderate (25%–50%), substantial (50%–75%), or considerable (>75%) (15). To test the robustness of our findings, we conducted sensitivity analyses by sequentially excluding individual trials and recalculating pooled estimates. Due to the small number of included trials in the meta-analyses (<10) (18), a formal assessment of publication bias using funnel plot asymmetry was not performed. All statistical computations were carried out using Review Manager (RevMan) version 5.4.1.

### Assessments of risk of bias and certainty of evidence

Risk of bias was assessed using the Cochrane Risk of Bias Tool Version 2 (RoB 2) (19), which includes domains such as the randomization process, intended interventions, missing outcome data, measurement of the outcome, and selection of the reported result. Each trial was assessed across these five bias domains, yielding both a summary risk-of-bias score for each domain and an overall classification (low risk, some concerns, or high risk of bias). Furthermore, two authors assessed each of the included studies, and each potential source of bias was graded as high, low, or unclear risk of bias, and two reviewers independently performed the assessment. Discrepancies were resolved by consensus or, if needed, through discussion with the research team.

The certainty of evidence was assessed using the Grading of Recommendations, Assessment, Development, and Evaluation (GRADE) approach (20). A total of two reviewers, both experienced in evidence synthesis, independently rated the quality of evidence across the five GRADE domains: risk of bias, inconsistency, indirectness, imprecision, and publication bias. In GRADE, all randomized clinical trials begin with a high rating and are downgraded based on risk of bias, inconsistency, indirectness, imprecision, or publication bias. Discrepancies in domain judgments or overall certainty were resolved through discussion and consensus, and persistent discordance was adjudicated by a third reviewer. Detailed GRADE rating criteria are shown in Supplementary material S2.

## Results

### Search results

Primary database searches yielded 6,084 unique articles for title and abstract screening. After removing duplicates, 4,249 articles were screened; 361 full-text articles were retrieved, of which 356 were excluded after evaluation. A manual search of other sources (e.g., backward and forward citation searches) identified 369 records, yielding one additional included article. Finally, following the inclusion criteria, six articles (12, 21–25) were considered eligible for inclusion in the meta-analysis.

### Study characteristics

In total, four RCTs reported across the six studies met the inclusion criteria, enrolling a total of 365 participants. The trials were conducted in Denmark (24, 25), Sweden (22, 23), and Canada (12, 21). Detailed descriptive characteristics are presented in Table 1. Reported mean ages by study group ranged approximately from 48.0 to 71.6 years across the trials. All included trials evaluated exercise-based prehabilitation delivered either as supervised outpatient sessions (12, 21–23) or home-based programs (24, 25) with therapist instructions. Common intervention elements across the trials included progressive strengthening and endurance exercises, spinal stabilization/motor-control training, individually tailored exercise prescriptions, and a behavioral/activation component in one study (23). Overall, the program dose most commonly comprised 2–3 sessions per week, with a total program duration of approximately 6–9 weeks. Control groups received usual care or standardized preoperative information and routine postoperative management. Pain and function were assessed using visual analog scales for back/leg pain (21, 23), the Oswestry Disability Index (ODI) (12, 21, 23), and the Roland–Morris Disability Questionnaire (RMDQ) in at least one trial (24). Health-related quality of life (HRQoL) was assessed using the European Quality of Life 5-Dimension (EQ-5D) (23) and the HRQoL-15D instrument (24). Psychological measures were reported using the Hospital Anxiety and Depression Scale (HADS) (23), the Beck Disability Index-Depression (12, 21), the Tampa Scale of Kinesiophobia (12, 21), and the Fear-Avoidance Beliefs Questionnaire-Physical Activity subscale (FABQ-PA) (23). Objective physical performance (knee extensor strength) (12, 21, 22) and health-service metrics (length of hospital stay) (12, 21, 24) were also reported in selected trials.

**Table 1:** Characteristics of the included studies.

Author (year), Country	Sample characteristics *N*; women(%); Age		Description of digital health interventions	Description of controls	Outcomes measures
	Intervention group	Control group			
Nielsen et al. (24, 25), Denmark	28; 61%; 48 (31–80)	32; 59%; 52 (23–88)	6–8 weeks home-based exercise + post-surgery intensified mobilization, balanced analgesia, protein supplements. Adherence rates: More than 80%. Adverse events: No adverse events were reported.	Routine information and standard postoperative care.	Back pain, Leg pain, Length of hospital stay, RMDQ, and HRQoL-15D
Lindbäck et al. (23), Fors et al. (22), Sweden	99; 54%; 58 (13.3)	98; 52%; 61 (11.5)	9-week physiotherapy (treatment-based manual therapy, motor control, or traction, tailored exercises, behavioral approach), 2 sessions/week. Adherence rates: 43 (43%) patients did not complete ≥12 treatment sessions for optimal adherence to treatment. Adverse events: No adverse events were reported.	Standardized surgical information and advice to stay active.	VAS back/leg pain, ODI, EQ-5D, FABQ-PA, HADS-Depression, and Knee extensor strength
Marchand et al. (21), Canada	20; 45%; 66.7 (11.6)	20; 40%; 71.5 (7.3)	6-week supervised exercise program (strength, endurance, spinal stabilization), 3 sessions/week, 30 min/session. Adherence rates: 88%. Adverse events: No adverse events were reported.	Standardized written pre-surgery information and routine hospital care.	ODI, VAS back/leg pain, Tampa Scale of Kinesiophobia, Beck Disability Index-Depression, Knee extensor strength, and Length of hospital stay
Marchand et al. (12), Canada	35; 40%; 66.2 (9.6)	33; 42%; 71.6 (7.6)	Supervised exercise sessions, 3 times/week for 6 weeks Adherence rates: More than 90.3%. Adverse events: No adverse events were reported.	Usual care	Back pain, Leg pain, ODI, Tampa Scale of Kinesiophobia, Beck Disability Index-Depression, Knee extensor strength, and Length of hospital stay

VAS, visual analog scale; ODI, Oswestry Disability Index; EQ-5D, European Quality of Life Five-Dimension questionnaire; FABQ-PA, Fear-Avoidance Beliefs Questionnaire-Physical Activity subscale; HADS-depression, hospital anxiety and depression scale-depression subscale; HRQoL-15D, health-related quality of life 15D instrument; RMDQ, Roland–Morris Disability Questionnaire.

### Risk-of-Bias assessment in individual studies

**Figure 2:**
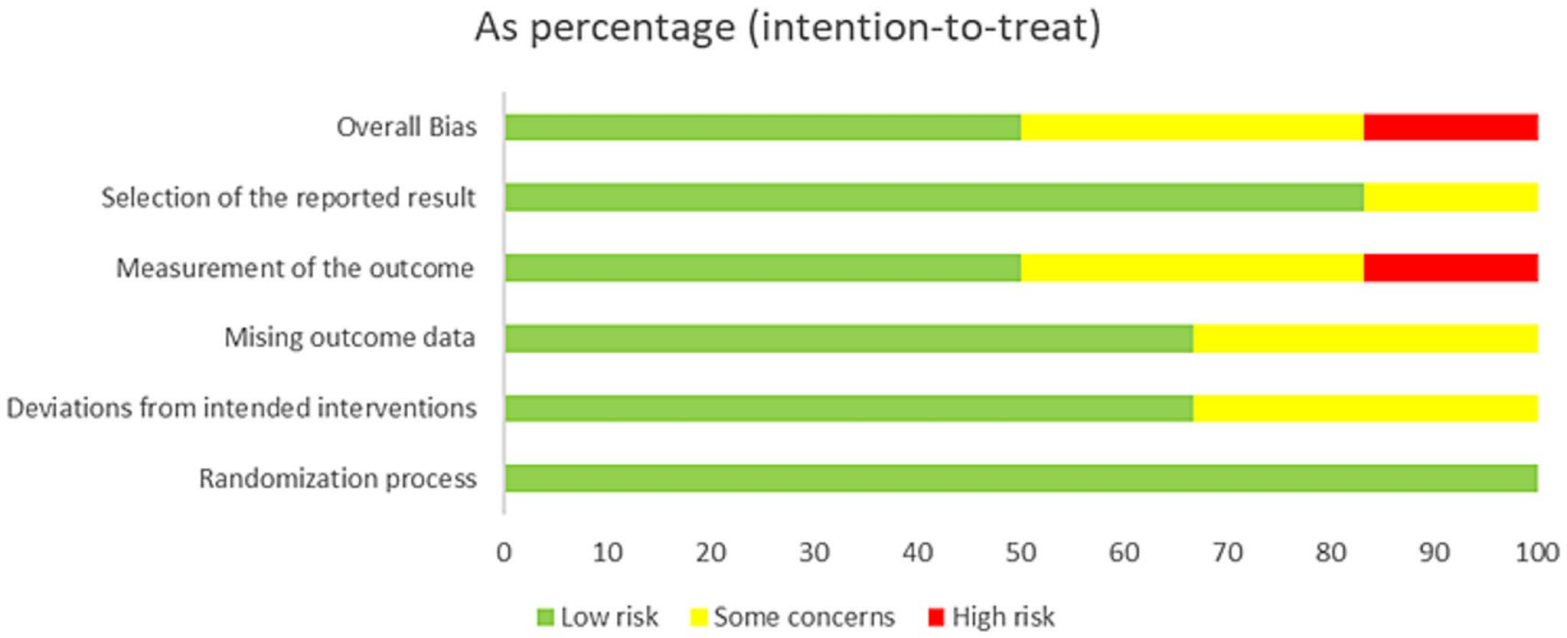
Risk of bias assessment for all included studies.

**Figure 3:**
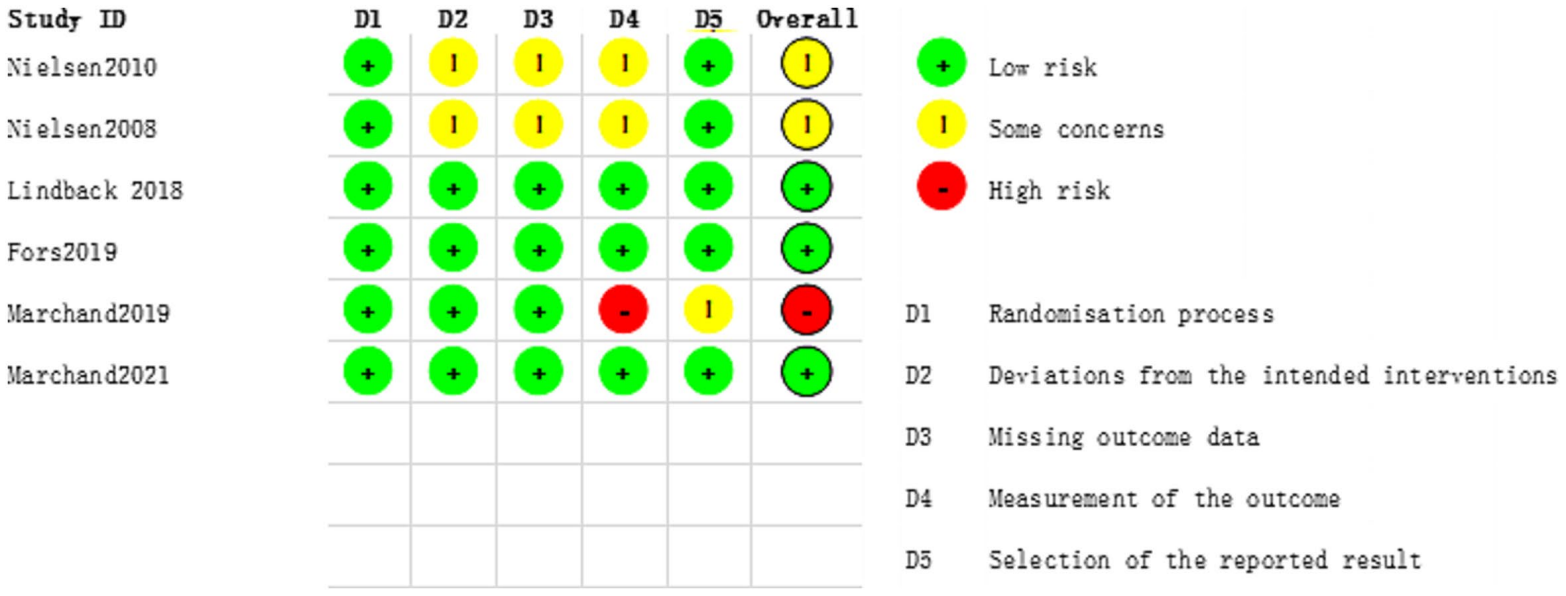
Summary of the distribution of different types of bias.

The assessment of risk of bias for all included trials is summarized in Figures 2, 3. Among the four included trials, two (50%) trials had low risk of bias (12, 23), one (25%) trial had high risk of bias (21), and one (25%) trial had some concerns of bias (24). The trial by Marchand et al. (21) was graded as high risk of bias because of the measurement of the outcomes.

### Effects of interventions

The GRADE evidence profiles summarizing the effects of exercise-based prehabilitation versus non-exercise controls on preoperative and postoperative outcomes are presented in Tables 2, 3, respectively.

**Table 2:** GRADE summary of findings for preoperative phase outcomes.

Exercise-based prehabilitation compared with non-exercise controls for patients undergoing elective spinal surgery					
Outcomes	Anticipated absolute effects^*^ (95% CI)		Relative effect (95% CI)	No of participants (studies)	Certainty of the evidence (GRADE)
	Risk with comparison group	Risk with intervention group			
Back pain	—	SMD **0.32 lower** (0.54 lower to 0.11 lower)	—	347 (4 RCTs)	⨁⨁⨁◯ Moderate^a^
leg pain	—	SMD **0.43 lower** (0.79 lower to 0.08 lower)	—	347 (4 RCTs)	⨁⨁⨁◯ Moderate^a^
Knee extensor strength	—	SMD **0.33 higher** (0.07 higher to 0.58 higher)	—	241 (3 RCTs)	⨁⨁⨁◯ Moderate^a^
Disability	—	SMD **0.44 lower** (0.65 lower to 0.23 lower)	—	364 (4 RCTs)	⨁⨁⨁◯ Moderate^a^
Kinesiophobia	—	SMD **0.3 lower** (0.53 lower to 0.07 lower)	—	287 (3 RCTs)	⨁⨁⨁◯ Moderate^a^
Depression	—	SMD **0.24 lower** (0.47 lower to 0.01 lower)	—	287 (3 RCTs)	⨁⨁⨁◯ Moderate^a^
Health-related quality of life	—	SMD **0.51 higher** (0.04 lower to 1.07 higher)	—	257 (2 RCTs)	⨁⨁⨁◯ Moderate^a^

*The risk in the intervention group (and its 95% confidence interval) is based on the assumed risk in the comparison group and the relative effect of the intervention (and its 95% CI).

CI, confidence interval; MD, mean difference; OR, odds ratio; SMD, standardized mean difference.

GRADE Working Group grades of evidence: *High certainty*: We are very confident that the true effect isclose to the estimated effect. *Moderate certainty*: We are moderately confident in the effect estimate: the true effect is likely close to the estimated effect, but there is a possibility that it is substantially different. *Low certainty*: Our confidence in the effect estimate is limited: the true effect may be substantially different from the estimated effect. *Very low certainty*: We have very little confidence in the effect estimate: the true effect is likely to be substantially different from the estimated effect.

a Total participants in the meta-analysis ≤400: downgrade by one level.The bold values means the risk in the intervention group.

**Table 3:** GRADE summary of findings for postoperative phase outcomes.

Exercise-based prehabilitation compared with non-exercise controls for patients undergoing elective spinal surgery					
Outcomes	Anticipated absolute effects^*^ (95% CI)		Relative effect (95% CI)	No of participants (studies)	Certainty of the evidence (GRADE)
	Risk with comparison group	Risk with intervention group			
Postoperative short-term phase (≤1 month)					
back pain	—	SMD **0.51 lower** (0.93 lower to 0.08 lower)	—	143 (3 RCTs)	⨁⨁⨁◯ Moderate^a^
Leg pain	—	SMD **0.17 lower** (0.93 lower to 0.6 higher)	—	143 (3 RCTs)	⨁⨁◯◯ Low^a^^,^^b^
Knee extensor strength	—	SMD **0.29 higher** (0.15 lower to 0.72 higher)	—	85 (2 RCTs)	⨁⨁⨁◯ Moderate^a^
Disability	—	SMD **0.03 lower** (0.42 lower to 0.36 higher)	—	162 (3 RCTs)	⨁⨁⨁◯ Moderate^a^
Kinesiophobia	—	SMD **0.01 higher** (0.42 lower to 0.43 higher)	—	87 (2 RCTs)	⨁⨁⨁◯ Moderate^a^
Depression	—	SMD **0.08 higher** (0.35 lower to 0.51 higher)	—	87 (2 RCTs)	⨁⨁⨁◯ Moderate^a^
Length of hospital stay (days)	The mean length of hospital stay (days) was **0**	MD **1.3 lower** (2.89 lower to 0.29 higher)	—	157 (3 RCTs)	⨁⨁◯◯ Low^a^^,^^b^
Postoperative Intermediate-term phase (1–6 months)					
Back pain	—	SMD **0.28 lower** (0.62 lower to 0.07 higher)	—	132 (3 RCTs)	⨁⨁⨁◯ Moderate^a^
Leg pain	—	SMD **0.18 higher** (0.16 lower to 0.52 higher)	—	132 (3 RCTs)	⨁⨁⨁◯ Moderate^a^
Disability	—	SMD **0.12 lower** (0.33 lower to 0.09 higher)	—	343 (4 RCTs)	⨁⨁⨁◯ Moderate^a^
Kinesiophobia	—	SMD **0.35 lower** (0.81 lower to 0.1 higher)	—	76 (2 RCTs)	⨁⨁⨁◯ Moderate^a^
Depression	—	SMD **0.07 lower** (0.31 lower to 0.17 higher)	—	273 (3 RCTs)	⨁⨁⨁◯ Moderate^a^
Health-related quality of life	—	SMD **0.13 higher** (0.23 lower to 0.49 higher)	—	253 (2 RCTs)	⨁⨁⨁◯ Moderate^a^
Postoperative long-term phase (≥6 months)					
Back pain	—	SMD **0.1 lower** (0.37 lower to 0.16 higher)	—	325 (4 RCTs)	⨁⨁⨁◯ Moderate^a^
Leg pain	—	SMD **0.02 lower** (0.24 lower to 0.2 higher)	—	325 (4 RCTs)	⨁⨁⨁◯ Moderate^a^
Disability	—	SMD **0.35 lower** (0.78 lower to 0.08 higher)	—	337 (4 RCTs)	⨁⨁⨁◯ Moderate^a^
Kinesiophobia	—	SMD **0.03 lower** (0.27 lower to 0.21 higher)	—	269 (3 RCTs)	⨁⨁⨁◯ Moderate^a^
Depression	—	SMD **0.27 lower** (0.72 lower to 0.18 higher)	—	269 (3 RCTs)	⨁⨁⨁◯ Moderate^a^
Health-related quality of life	—	SMD **0.04 lower** (0.3 lower to 0.22 higher)	—	253 (2 RCTs)	⨁⨁⨁◯ Moderate^a^

*The risk in the intervention group (and its 95% confidence interval) is based on the assumed risk in the comparison group and the relative effect of the intervention (and its 95% CI).

CI, confidence interval; MD, mean difference; OR, odds ratio; SMD, standardized mean difference.

GRADE Working Group grades of evidence: *High certainty*: We are very confident that the true effect is close to the estimated effect. *Moderate certainty*: We are moderately confident in the effect estimate: the true effect is likely close to the estimated effect, but there is a possibility that it is substantially different. *Low certainty*: Our confidence in the effect estimate is limited: the true effect may be substantially different from the estimated effect. *Very low certainty*: We have very little confidence in the effect estimate: the true effect is likely to be substantially different from the estimated effect.

a Total participants in the meta-analysis ≤400: downgrade by one level.

b *I*^2^ > 75% (serious heterogeneity): downgrade by one level.The bold values means the risk in the intervention group.

### Preoperative phase (after prehabilitation)

**Figure 4:**
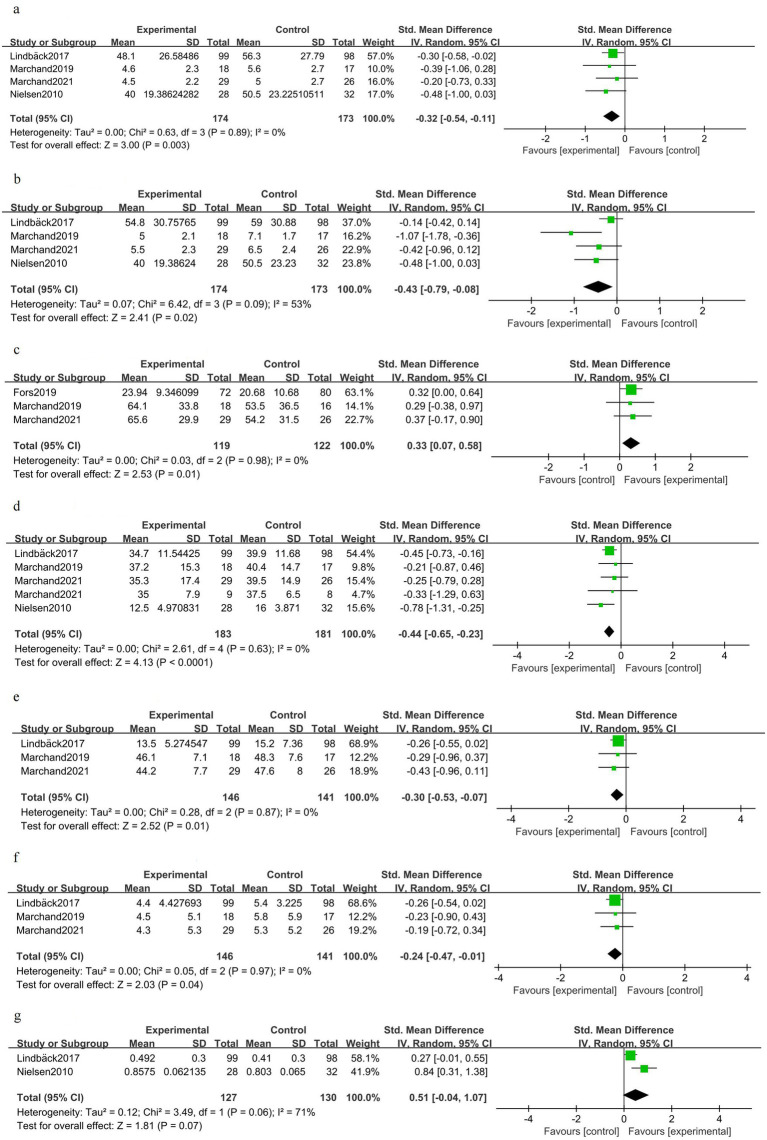
Forest plot for the preoperative phase (after prehabilitation).

Back pain was slightly reduced after exercise-based prehabilitation compared to controls [SMD −0.32 (95% CI − 0.54 to −0.11); four trials, 347 patients; *I*^2^ 0%; GRADE: Moderate] (Figure 4a). Leg pain favored prehabilitation to a small–moderate degree [SMD −0.43 (95% CI − 0.79 to −0.08); four trials, 347 patients; *I*^2^ 53%; GRADE: Moderate] (Figure 4b). Knee extensor strength was greater in the prehabilitation group [SMD 0.33 (95% CI 0.07–0.58); three trials, 241 patients; *I*^2^ 0%; GRADE: Moderate] (Figure 4c). Disability was reduced following prehabilitation [SMD −0.44 (95% CI −0.65 to −0.23); four trials, 364 patients; *I*^2^ 0%; GRADE: Moderate] (Figure 4d). Fear of movement (kinesiophobia) was modestly lower after prehabilitation [SMD 0.30 (95% CI −0.53 to −0.07); three trials, 287 patients; *I*^2^ 0%; GRADE: Moderate] (Figure 4e). Depressive symptoms showed a small improvement with prehabilitation [SMD 0.24 (95% CI −0.47 to −0.01); three trials, 287 patients; *I*^2^ 0%; GRADE: Moderate] (Figure 4f). Health-related quality of life favored prehabilitation in the pooled estimate, but the confidence intervals included no effect [SMD 0.51 (95% CI −0.04 to 1.07); two trials, 257 patients; *I*^2^ 71%; GRADE: Moderate] (Figure 4g).

### Postoperative short-term phase (≤1 month)

**Figure 5:**
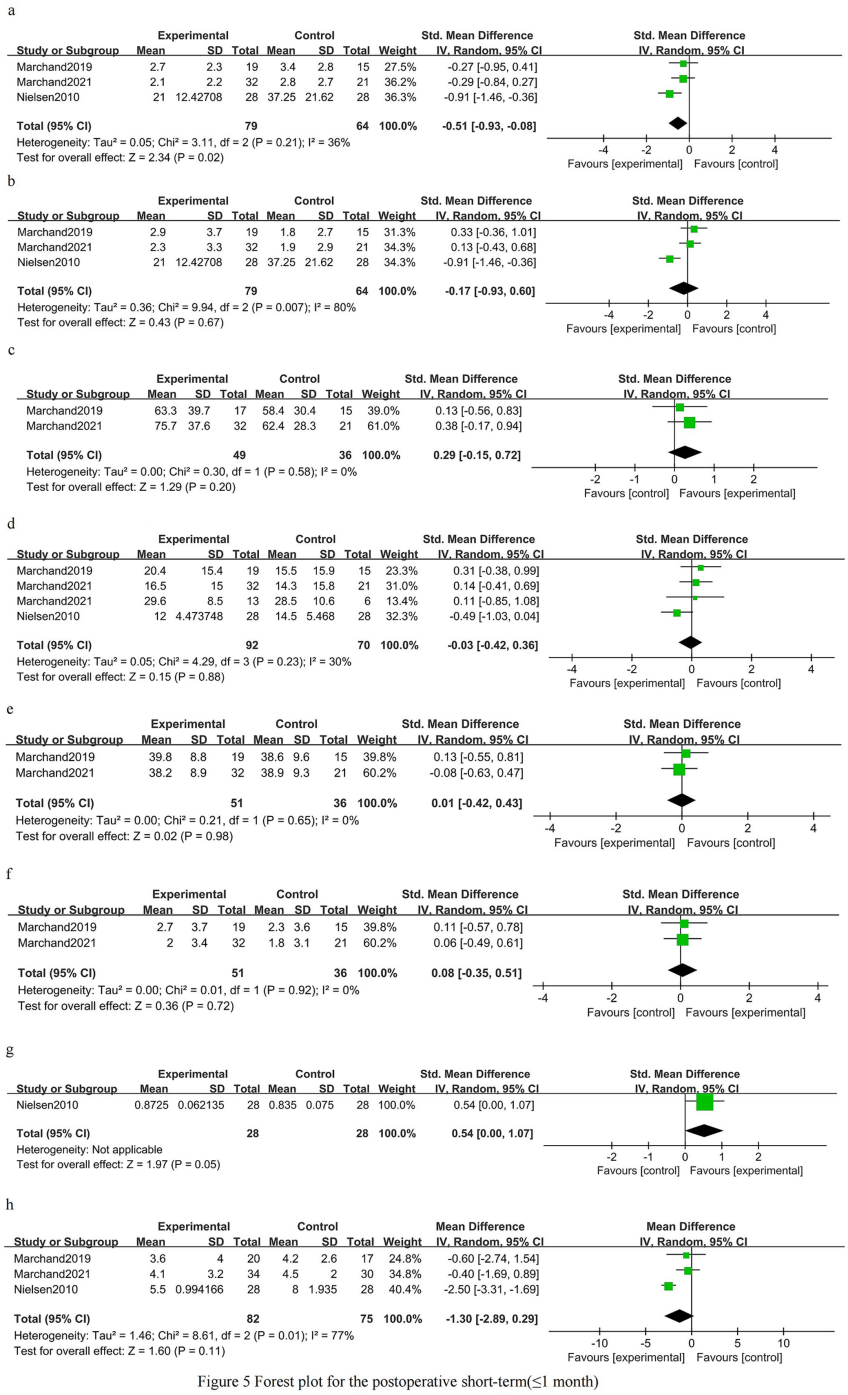
Forest plot for the postoperative short-term phase (≤1 month).

Back pain in the early postoperative period was improved in patients who received prehabilitation [SMD −0.51 (95% CI −0.93 to −0.08); three trials, 143 patients; *I*^2^ 36%; GRADE: Moderate] (Figure 5a). There was no clear early postoperative benefit for leg pain [SMD −0.17 (95% CI −0.93 to 0.60); three trials, 143 patients; *I*^2^ 80%; GRADE: Low] (Figure 5b). Early postoperative knee extensor strength showed a non-significant trend favoring prehabilitation [SMD 0.29 (95% CI −0.15 to 0.72); two trials, 85 patients; *I*^2^ 0%; GRADE: Moderate] (Figure 5c). Overall disability scores did not differ significantly in the early postoperative period [SMD −0.03 (95% CI −0.42 to 0.36); three trials, 162 patients; *I*^2^ 30%; GRADE: Moderate] (Figure 5d). Kinesiophobia was unchanged shortly after surgery [SMD 0.01 (95% CI −0.42 to 0.43); two trials, 87 patients; *I*^2^ 0%; GRADE: Moderate] (Figure 5e). Depressive symptoms showed no clear early postoperative difference [SMD 0.08 (95% CI −0.35 to 0.51); two trials, 87 patients; *I*^2^ 0%; GRADE: Moderate] (Figure 5f). One trial reported improved early postoperative health-related quality of life [SMD 0.54 (95% CI 0.00–1.07); one trial, 56 patients] (Figure 5g). Length of hospital stay was shorter on average in the prehabilitation group, but the difference did not reach statistical significance [MD −1.30 days (95% CI −2.89 to 0.29); three trials, 157 patients; *I*^2^ 77%; GRADE: Low] (Figure 5h).

### Postoperative intermediate-term phase (1–6 months)

**Figure 6:**
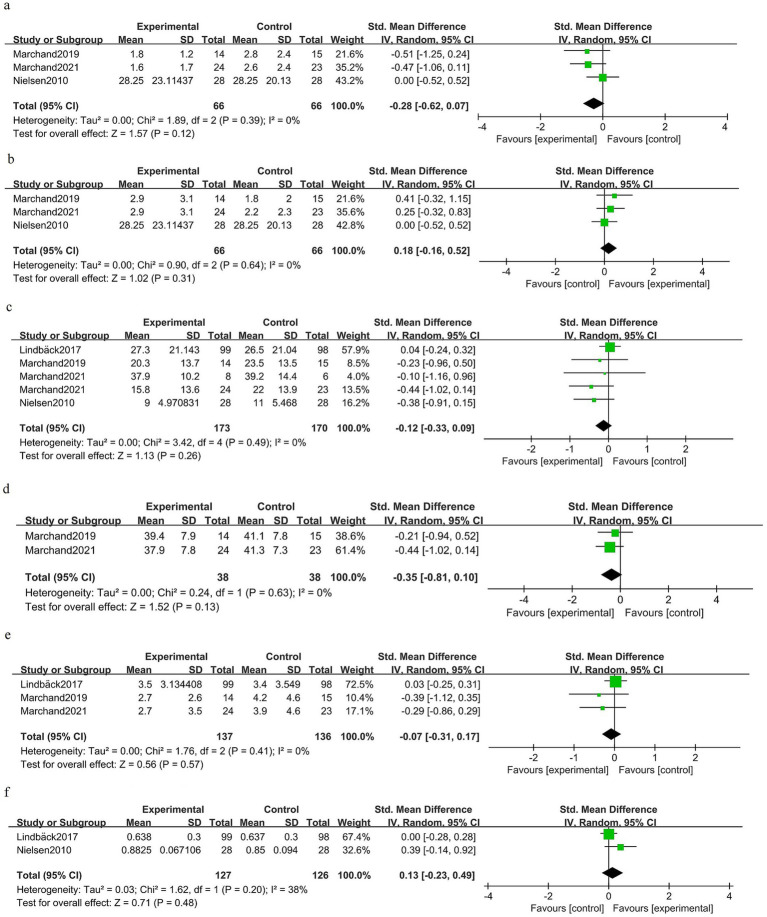
Forest plot for the postoperative intermediate-term phase (1–6 months).

At 1–6 months after surgery, there was no clear benefit of prehabilitation for back pain [SMD −0.28 (95% CI −0.62 to 0.07); three trials, 132 patients; *I*^2^ 0%; GRADE: Moderate] (Figure 6a). Leg pain at 1–6 months was similar between the groups [SMD 0.18 (95% CI −0.16 to 0.52); three trials, 132 patients; *I*^2^ 0%; GRADE: Moderate] (Figure 6b). Disability measured in the intermediate postoperative period did not differ significantly between the groups [SMD −0.12 (95% CI −0.33 to 0.09); four trials, 343 patients; *I*^2^ 0%; GRADE: Moderate] (Figure 6c). Kinesiophobia showed a non-significant difference favoring prehabilitation [SMD −0.35 (95% CI −0.81 to 0.10); two trials, 76 patients; *I*^2^ 0%; GRADE: Moderate] (Figure 6d). Depressive symptoms were not significantly different at 1–6 months [SMD −0.07 (95% CI −0.31 to 0.17); three trials, 273 patients; *I*^2^ 0%; GRADE: Moderate] (Figure 6e). Health-related quality of life did not differ at intermediate follow-up [SMD 0.13 (95% CI −0.23 to 0.49); two trials, 253 patients; *I*^2^ 38%; GRADE: Moderate] (Figure 6f).

### Postoperative long-term phase (≥6 months)

**Figure 7:**
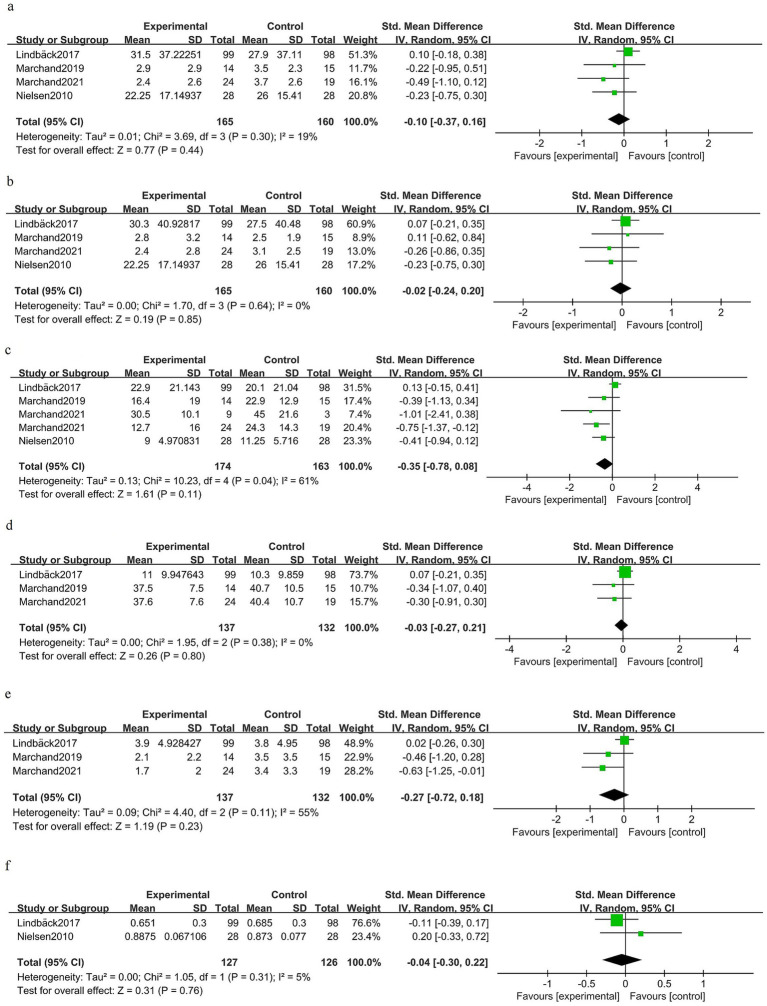
Forest plot for the postoperative long-term phase (≥6 months).

At ≥6 months after surgery, there was no significant difference in back pain between the groups [SMD −0.10 (95% CI −0.37 to 0.16); four trials, 325 patients; *I*^2^ 19%; GRADE: Moderate] (Figure 7a). Long-term leg pain outcomes were similar for prehabilitation and control groups [SMD −0.02 (95% CI −0.24 to 0.20); four trials, 325 patients; *I*^2^ 0%; GRADE: Moderate] (Figure 7b). Pooled estimates for long-term disability did not demonstrate a statistically significant benefit [SMD −0.35 (95% CI −0.78 to 0.08); four trials, 337 patients; *I*^2^ 61%; GRADE: Moderate] (Figure 7c). Kinesiophobia at long-term follow-up was unchanged [SMD − 0.03 (95% CI −0.27 to 0.21); three trials, 269 patients; *I*^2^ 0%; GRADE: Moderate] (Figure 7d). Depressive symptoms at ≥6 months showed no clear benefit of prehabilitation [SMD −0.27 (95% CI −0.72 to 0.18); three trials, 269 patients; *I*^2^ 55%; GRADE: Moderate] (Figure 7e). Health-related quality of life at late follow-up did not differ between the groups [SMD −0.04 (95% CI −0.30 to 0.22); two trials, 253 patients; *I*^2^ 5%; GRADE: Moderate] (Figure 7f).

## Discussion

In this systematic review and meta-analysis of randomized controlled trials, exercise-based prehabilitation before elective spinal surgery produced modest but directionally consistent improvements in several preoperative patient-reported and performance outcomes, while early and later postoperative effects were smaller and less consistent. The overall certainty of the evidence was low to moderate, owing to imprecision and/or inconsistency.

Preoperative outcomes favored exercise-based prehabilitation, supported by moderate-quality evidence, including reductions in back and leg pain, improved lower limb muscle strength, and modest improvements in disability, kinesiophobia, and depressive symptoms. In the early postoperative period, there was moderate-quality evidence, which indicated that exercise-based prehabilitation reduces back pain, whereas most other early outcomes—including leg pain, knee extensor strength, disability, kinesiophobia, depression, and health-related quality of life—showed small or imprecise effects with moderate-quality evidence. In addition, low-quality evidence suggested that exercise-based prehabilitation may reduce length of hospital stay by 1.3 days [−2.89, 0.29]; however, the result should be interpreted with caution since the confidence intervals also include the null effect. By intermediate- and long-term follow-up (1–6 months and ≥6 months), pooled estimates generally clustered closer to the null and failed to demonstrate clear, durable advantages across the majority of patient-centered outcomes, although the direction of effect in multiple domains continued to favor prehabilitation in pooled standardized metrics. Importantly, the absence of convincing statistical significance for some postoperative outcomes should not be interpreted as definitive evidence of no effect. The limited number and size of trials, variable intervention fidelity, and clinical heterogeneity mean that current data are insufficient to rule out clinically important benefits under optimized conditions.

### Limitations

Several factors limit confidence in our conclusions. First, most included randomized trials were small and often single-center, limiting statistical power to detect modest but clinically meaningful postoperative effects. Second, adherence and fidelity reporting were inconsistent across the studies, restricting the ability to evaluate dose–response relationships and to determine whether null or small effects reflect inadequate exposure rather than true ineffectiveness. Third, outcome measurement varied across the studies (e.g., different pain and disability instruments and follow-up timings), requiring pooling via standardized metrics that enhance comparability but can obscure clinically interpretable absolute differences on familiar scales. Fourth, blinding is inherently difficult in exercise interventions, and the lack of participant and provider blinding may bias subjective outcomes despite randomized allocation. In addition, many studies relied on self-reported measures, while objective outcomes were reported in fewer trials (26), both of which are prone to bias. Finally, generalizability is limited because many trials enrolled selected surgical populations with few comorbidities, leaving unanswered how prehabilitation performs in older, frailer, or socioeconomically diverse patients who represent substantial proportions of real-world spine surgical cohorts (27, 28).

### Future considerations

Given that standardized effect estimates often favored prehabilitation despite a lack of consistent statistical significance, future studies should adopt a pragmatic and hypothesis-driven approach to determine when, for whom, and how prehabilitation produces clinically meaningful and durable benefit. First, adequately powered, multicenter randomized trials are required that compare clearly specified exercise protocols with prespecified intensity, frequency, and supervision and include rigorous adherence and fidelity monitoring to enable dose–response analyses and per-protocol assessment (29). Second, trials should prespecify and harmonize core outcome sets and evaluate effects at standardized time points to enable meaningful temporal synthesis and GRADE-based certainty appraisal. Third, investigators should evaluate effect modification by plausible patient and surgical characteristics (baseline pain severity, frailty or sarcopenia, psychological comorbidity such as kinesiophobia or depression, and procedure type/invasiveness) to identify subgroups most likely to benefit and thereby enable targeted, cost-effective deployment (3, 30). Fourth, mechanistic and mediation studies embedded within trials—for example, neuromuscular control assessments, biomarkers of inflammation or central sensitization, and measurement of behavioral mediators—would help clarify causal pathways and identify intermediate markers predictive of sustained benefit (31). Fifth, given the multimodal nature of surgical risk, trials comparing exercise-only prehabilitation with multimodal programs (exercise plus nutrition, smoking cessation, and psychological interventions) will be important to determine whether synergistic interventions produce larger or more durable postoperative improvements (32). Sixth, economic evaluations should be embedded in future trials to determine value—whether modest preoperative gains translate into reduced postoperative resource use, faster return to function, or favorable cost-utility metrics in routine care. Finally, implementation research exploring scalable delivery models (13, 33) (supervised, home-based, hybrid, or telehealth), equitable access, integration within Enhanced Recovery After Surgery (ERAS) or primary care workflows, and acceptability across diverse healthcare settings will be essential to translate prehabilitation into routine practice where it can be most effective. (34, 35). Therefore, exercise-based prehabilitation should receive greater attention and utilization among spinal physicians. This is both consistent with the clinical practice of ERAS in spinal surgery and more conducive to patients’ functional recovery.

## Conclusion

Exercise-based prehabilitation for adults undergoing elective spinal surgery produces consistent small-to-moderate standardized benefits across most preoperative domains and shows a favorable signal for early postoperative back pain; however, evidence of sustained intermediate- and long-term postoperative improvement is not established with current data. Future large, well-designed trials that harmonize outcomes, clarify optimal intervention components and dosing, identify responder subgroups, include mechanistic and economic endpoints, and evaluate scalable delivery models are urgently needed to determine whether exercise-based prehabilitation can produce durable improvements in recovery after spine surgery and to guide evidence-based implementation.

## Data Availability

The original contributions presented in the study are included in the article/Supplementary material, further inquiries can be directed to the corresponding author.
