# Supplementary material for: Efficacy of exercise-based prehabilitation for patients undergoing elective spinal surgery: a systematic review and meta-analysis
Source: Front Med (Lausanne). 2025 Nov 19;12:1707138. doi: 10.3389/fmed.2025.1707138 (PMC12673885; doi:10.3389/fmed.2025.1707138)
Supplement: Supplementary file 1 [file Table_1.docx]

**Additional file 1. Example search strategy.**

The search strategy for PubMed was shown below. It was adapted for the other databases.

| **Pubmed**  ("Preoperative Exercise"[MeSH Terms] OR "preoperative exercis*"[Title/Abstract] OR "pre operative exercis*"[Title/Abstract] OR "Preoperative Conditioning"[Title/Abstract] OR "pre operative conditioning"[Title/Abstract] OR "Preoperative Rehabilitation"[Title/Abstract] OR "pre operative rehabilitation"[Title/Abstract] OR "Prehabilitation"[Title/Abstract] OR "prehabilit*"[Title/Abstract] OR "pre habilit*"[Title/Abstract] OR "pre habilitat*"[Title/Abstract] OR (("exercis*"[Title/Abstract] OR "stretch*"[Title/Abstract] OR "aerobic*"[Title/Abstract] OR "physical activit*"[Title/Abstract] OR "Exercise Therapy"[MeSH Terms] OR "Exercise"[MeSH Terms] OR "Physical Fitness"[MeSH Terms]) AND ("Preoperative Care"[MeSH Terms] OR "Preoperative Period"[MeSH Terms] OR "preoperat*"[Title] OR "pre operat*"[Title] OR "presurg*"[Title] OR "pre surg*"[Title]))) AND ("randomized controlled trial"[Publication Type] OR "random*"[Title/Abstract] OR "placebo"[Title/Abstract]) | **Adapted for:**  Cochrane, Embase,  Web of Science, and Scopus. |
| --- | --- |
