# Supplementary material for: Efficacy of exercise-based prehabilitation for patients undergoing elective spinal surgery: a systematic review and meta-analysis
Source: Front Med (Lausanne). 2025 Nov 19;12:1707138. doi: 10.3389/fmed.2025.1707138 (PMC12673885; doi:10.3389/fmed.2025.1707138)
Supplement: Supplementary file 2 [file Table_2.docx]

**Additional file 2. Detailed GRADE rating criteria：**

1. **Risk of bias**: If half of the study participants derive from trials judged at high risk of bias, we downgrade the certainty by one level.
2. **Inconsistency**:
   - I² > 75% (serious heterogeneity): downgrade by one level
3. **Imprecision**:
   - Total participants in the meta-analysis ≤ 400: downgrade by one level
4. **Publication bias**: Detection of bias via statistical tests leads to a one-level downgrade. If there were not able to examine publication bias, we did not downgrade for publication bias.
5. **Indirectness**: When over 50% of participants, interventions, comparators, or outcomes do not directly match the review’s eligibility criteria, we lower the certainty by one level.
